# Characterization of clinical extensively drug-resistant *Pseudomonas aeruginosa* in the Hunan province of China

**DOI:** 10.1186/s12941-016-0148-y

**Published:** 2016-05-23

**Authors:** Jun Li, Mingxiang Zou, Qingya Dou, Yongmei Hu, Haichen Wang, Qun Yan, Wen’ en Liu

**Affiliations:** Department of Clinical Laboratory, Xiangya Hospital, Central South University, No. 87, Xiangya Road; Kaifu District, Changsha, 410008 Hunan China; Department of Infection Control Center, Xiangya Hospital, Central South University, Changsha, 410008 Hunan China

**Keywords:** Extensively drug-resistant *Pseudomonas aeruginosa*, Resistance mechanism, Movable genetic elements, Molecular epidemiology, PFGE

## Abstract

**Background:**

*Pseudomonas aeruginosa* strains that are classed as extensively drug resistant (XDR-PA) are resistant to all antibiotics except for one or two classes and are frequently the cause of hard-to-treat infections worldwide. Our study aimed to characterize clinical XDR-PA isolates recovered during 2011–2012 at nine hospitals in the Hunan province of China.

**Methods:**

Thirty-seven non-repetitive XDR-PA strains from 37 patients were investigated for genes encoding antimicrobial resistance determinants, efflux pumps, outer membrane proteins, and movable genetic elements using polymerase chain reaction (PCR). The expression of genes encoding the efflux pump component MexA and the outer membrane protein OprD was measured using real-time PCR. In addition, clonal relatedness of these XDR-PA isolates was analyzed by pulsed-field gel electrophoresis (PFGE).

**Results:**

Various genes encoding antimicrobial resistance determinants were found in all isolates. In particular, the *bla*_TEM-1_, *bla*_CARB_, *armA*, *bla*_IMP-4_, *bla*_VIM-2_, and *rmtB*, were found in 100, 37.8, 22, 22, 19 and 5 % of the isolates, respectively. Remarkably, two isolates coharbored *bla*_IMP-4_, *bla*_VIM-2_, and *armA*. In all 37 antibiotic-resistant strains, the relative expression of *oprD* was decreased while *mexA* was increased compared to the expression of these genes in antibiotic-susceptible *P. aeruginosa* strains. All of the XDR-PA isolates harbored class I integrons as well as multiple other mobile genetic elements, such as *tnpU*, *tnp513*, *tnpA* (Tn*21*), and *merA*. A high genotypic diversity among the strains was detected by PFGE.

**Conclusions:**

Multiple antibiotic-resistance mechanisms contributed to the drug resistance of the XDR-PA isolates investigated in this study. Thus, the XDR-PA isolates in this area were not clonally related. Instead, multiple types of movable genetic elements were coharbored within each XDR-PA isolate, which may have aided the rapid development of these XDR-PA strains. This is the first report of XDR-PA strains that coharbor *bla*_IMP-4_, *bla*_VIM-2_, and *armA*.

## Background

*Pseudomonas aeruginosa*, a rod-shaped, non-fermenting gram-negative bacterium, causes nosocomial infections that can lead to sepsis, pneumonia, endocarditis, and urinary tract infections. The emergence of extensively drug-resistant *P. aeruginosa* (XDR-PA) strains showing resistant to all antimicrobial agents except for one or two classes is becoming a major public health concern [1–5].

That was found by previous studies the mechanisms of antibiotic resistance associated with clinical XDR-PA isolates are complex [6, 7]. The prevailing hypothesis is that XDR-PA isolates acquire numerous drug-resistance determinants through horizontal gene transfer that is mediated by mobile genetic elements [8–10]. In addition, genes encoding the antibiotic-inactivating enzymes β-lactamases, aminoglycoside-modifying enzymes (AMEs), and 16S rRNA methylases (16S-RMTases) are frequently associated with antibiotic resistance in XDR-PA strains. In recent studies, an over-expression of drug-efflux pumps and diminished expression of outer membrane proteins are suggested to play a role in drug resistance [11–14].

Although the prevalence of drug-resistance determinants in XDR-PA strains isolated in other countries has been determined [15, 16], few studies have investigated the resistance mechanisms and the epidemiological profiles of clinical XDR-PA isolates found in China. Before 2011, XDR-PA strains were rarely found in China; thereafter, a gradual emergence has occurred in some hospitals. Therefore, the purpose of this study was to analyze the resistance mechanisms and molecular epidemiology of clinical XDR-PA strains isolated earlier in our region recovered from 2011 to 2012.

## Methods

### Bacterial isolates

Thirty-seven out of 482 (7.7 %) *P. aeruginosa* isolates that were screened were XDR-PA strains displaying resistance to all antimicrobial agents except colistin. The *P. aeruginosa* strains were isolated from September 1, 2011 to June 30, 2012 from nine of fifteen different hospitals in the Hunan province of China. Drug susceptibility of XDR-PA isolates was determined by the Kirby-Bauer (K-B) disk diffusion method and two quality control strains (*Escherichia coli* ATCC25922 and *P. aeruginosa* ATCC27853) were included in the analyses. The results were analyzed and interpreted according to the guidelines of Clinical and Laboratory Standards Institute (CLSI) [17]. To prevent analysis of redundant strains, only the first strain was collected when duplicate strains were from the same patient.

### Phenotypic tests for carbapenemase production

The modified Hodge test (MHT) was used to detect the production of carbapenemase using an imipenem disc (10 μg) as described by CLSI [17]. A combined-disc test was carried out to detect the production of metallo-β-lactamase (MBL). Two discs [One disc contained imipenem (10 μg) and 5 μL of 0.5 M EDTA (Sigma Chemicals), and the other disc contained only imipenem (10 μg)] were placed 20 mm apart on a Mueller–Hinton agar plate inoculated with each test strain. A strain was considered positive for metallo-β-lactamase production when the zone diameter around the imipenem-EDTA disc was more than 4 mm of the imipenem-only disc [18].

### Detection of genes encoding antimicrobial resistance determinants and genes associated with movable genetic elements

The DNA templates used in polymerase chain reactions (PCR) to amplify genes encoding antimicrobial resistance determinants were obtained as follows: bacterial suspensions were incubated for 10 min at 95 °C followed by centrifugation at 10,000×*g* for 10 min to remove cellular debris. Genes coding for carbapenem β-lactamases (*bla*_KPC_, *bla*_SME_, *bla*_GES_, *bla*_IMI_/*bla*_NMC_, *bla*_NDM-1_, *bla*_VIM-2_, *bla*_IMP-4_, *bla*_SIM-1_, *bla*_GIM_, *bla*_SPM_, *bla*_OXA-23_, and *bla*_OXA-51_), extended-spectrum β-lactamases (ESBLs) (*bla*_TEM-1_, *bla*_CTX-M_, *bla*_SHV_, *bla*_OXA-1_, *bla*_OXA-2_, *bla*_OXA-10_, *bla*_VEB_, and *bla*_PER_), and AmpC β-lactamases (*bla*_MOX_, *bla*_FOX_, *bla*_DHA_, *bla*_CIT_, and *bla*_EBC_) were performed by PCR with previously described primers [18–20]. In addition, the isolates were screened by PCR for AME genes (*aac(3)*-*IIa* and *ant(2′’)*-*Ia*) [21], 16S-RMTases (*armA*, *npmA*, *rmtA*, *rmtB*, *rmtC*, *rmtD*, and *rmtE*) [22], a drug-efflux pump component (*mexA*), and an outer membrane protein (*oprD*). The PCR primers used to screen for these genes are listed in Table 1. To detect genes associated with the movable genetic elements, *intI*, *traA*, *traF*, *trbC*, *tnp513*, IS*pa7*, IS*Ecp1*, *tnpU*, *tnpA* (Tn*21*), *tnsA*, and *merA,* PCR was performed with primers shown in Table 1. All amplified DNA fragments were sequenced and then analyzed using the BLAST program (http://www.ncbi.nlm.nih.gov/BLAST).

**Table 1:** Primers used in this study for PCR and RT-PCR analyses

Gene	Sequence (5′–3′)	Fragment length (bp)
*intI*	F:CCGAGGATGCGAACCACTTC R:CCGCCACTGCGCCGTTACCA	789
*traA*	F:AAGTGTTCAGGGTGCTTCTGCGC R:GTCATGTACATGATGACCATTT	272
*traF*	F:CGGTGATGATTTGCGAACGA R:AGCATTCCGGTCGGCCTGTA	400
*trbC*	F:CGGYATWCCGSCSACRCTGCG R:GCCACCTGYSBGCAGTCMCC	255
*tnp513*	F:ATGTCGCTGGCAAGGAACGC R:GGGTTCGCTGCGAGGATTGT	240
IS*pa7*	F:TCAGGCCTTCATCGCTGCCATCAGG R:TAGGCGTACAGTGCTCTTTCAACGCA	300
IS*Ecp1*	F:CTTCATTGGCATTGATAAGTTAG R:TGTAGCATCGGTTTCCCAGTTTC	299
*tnpU*	F:CCAACTGATGGCGGTGCCTT R:CGGTATGGTGGCTTTCGC	403
*tnpA* *(*Tn*21*)	F:ATGCCACGTCGTTCCATCCTGTCC R:CCGGGTCTGCTCCCGCTGGCC	300
*tnsA*	F:GCAGCAGCCTTACAAGACGAG R:GCCACATAGCGCAACTCCTCC	416
*merA*	F:GACCAGCCGCAGTTCGTCTA R:GCAGCASGAAAGCT GCTTCA	462
*mexA*	F:CGACCAGGCCGTGAGCAAGCAGC R:GGAGACCTTCGCCGCGTTGTCGC	275
*oprD*	F:ATGAAAGTGATGAAGTGGAGCG R:TTACAGGATCGACAGCGGATAG	949
*mexA* in RT-PCR	F:GGCGACAACGCGGCGAAGG R:CCTTCTGCTTGACGCCTTCCTGC	202
*oprD* in RT-PCR	F:CGGCGACATCAGCAACACC R:GGGCCGTTGAAGTCGGAGTA	195
*16S rRNA* in RT-PCR	F:CCTACGGGAGGCAGCAG R:ATTACCGCGGCTGCTGG	194

### Quantifying expression of *mexA* and *oprD*

The expression of *mexA* and *oprD* in the XDR-PA isolates was determined by real-time PCR (RT-PCR). The experimental group consisted of the 37 XDR-PA isolates and the control group was made up of 31 *P. aeruginosa* isolates that were collected at the same time as the XDR-PA strains but were shown to be sensitive to all antimicrobial agents tested. The primers used for quantifying *mexA* and *oprD* expression are listed in Table 1. The RT-PCR reactions were carried out by a QuantiFast SYBR Green RT-PCR Kit from Qiagen and a Real-Time PCR System of LightCycler 2.0 from Roche, Burgess Hill, UK, according to the manufacturer protocols. All reactions were repeated three times using 10 ng of RNA template that was prepared using the RNeasy Mini Kit from Qiagen, Crawley, UK and treated with Dnase. Gene expression was normalized relative to that of the *16S rRNA* gene using the 2^−△△CT^ method [23]. The expression of *16S rRNA* gene was determined by the primers shown in Table 1.

### Pulsed-field gel electrophoresis (PFGE)

Clonal relatedness of the XDR-PA isolates was analyzed by PFGE. Preparation of genomic DNA was done in agarose blocks. The DNA was then digested by the restriction enzyme *Xba*I from Promega, USA, followed by embedding into 1 % PFGE agarose gel. PFGE was performed for 24 h using the GenePath System from Bio-Rad with the follow conditions: 5.5 V/cm, 12 °C, 120°, and a switch time from 4 to 40 s. The molecular size marker, *Salmonella enterica* strain H9812, was obtained from the respiratory laboratory of infectious diseases, CDC, China. The gel was stained for 30 min with ethidium bromide and the gel image was documented using the gel documentation system Gel Doc 2000 from Bio-Rad. Finally, analyses of the results were performed by the BioNumerics software platform (Applied Math, Sint-Maten-Latem, Belgium) and visual inspection with the criteria of relatedness proposed by Tenover et al. [24].

### Statistical analysis

WHONET software (version 5.4, WHO) was used to analyze the patient demographic information and the antibiotic resistance data using the 2014 CLSI criteria for breakpoints for *P. aeruginosa*. Comparisons of different groups were analyzed by a two-sided Chi square (*χ*^*2*^) test with the SPSS13.0 software (SPSS Inc., USA). A *P* < 0.05 was regarded as statistically significant.

## Results

### Phenotypic screening and resistance determinants

**Fig. 1:**
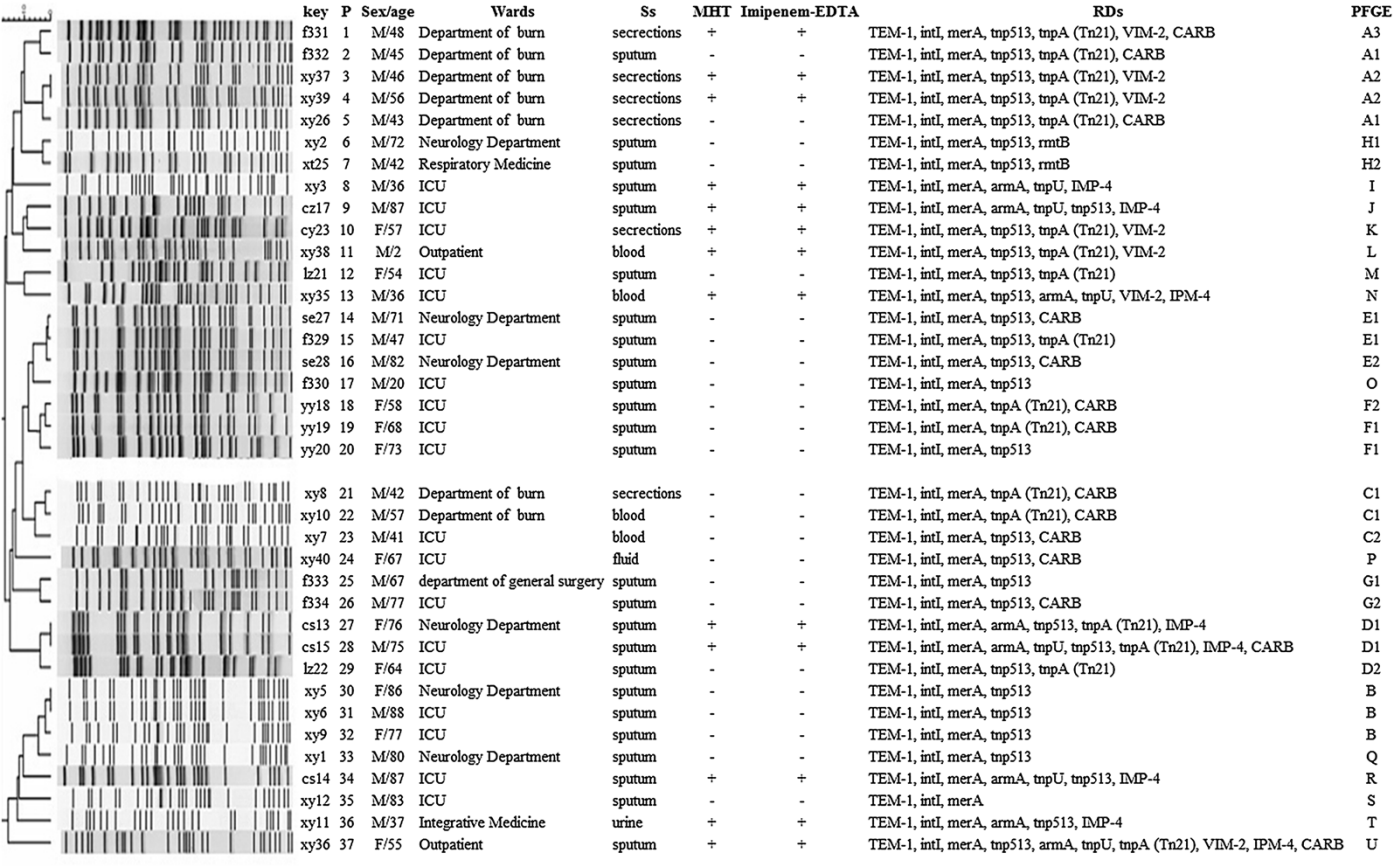
Clinical features, molecular characterization, and dendrogram based on PFGE of 37 XDR-PA isolates. The dendrogram was developed using the BioNumerics software platform. *P* patients, *Ss* specimens, *RDs* resistance determinants, *xy* Xiangya Hospital, *f*_*3*_ the Third Xiangya Hospital, *yy* People’s Hospital of Liuyang, *cs* the Third Hospital of Changsha, *se* the Second People’s Hospital of Hunan province, *xt* Xiangtan Central Hospital, *lz* Chinese Medicine Hospital of Liuyang, *cz* People’s Hospital of Chenzhou, *cy* the First Hospital of Changsha, and *A*–*U* PFGE types

Of the 37 XDR-PA isolates, 13 (35.1 %) isolates were positive for MTH and MBL, while all other isolates were negative (Fig. 1).

Diverse types of genes encoding antibiotic-inactivating enzymes were detected. Specifically, the genes *bla*_TEM-1_, *bla*_CARB_, *armA*, *bla*_IMP-4_, *bla*_VIM-2_, and *rmtB* were found in 100, 37.8, 22, 22, 19 and 5 % of the XDR-PA isolates, respectively. The ESBL gene *bla*_TEM-1_ was found in all isolates, while the other ESBL genes that were tested were not found in any of the isolates. Among the 37 XDR-PA isolates, 13 (35.1 %) isolates were positive for MBLs genes, 8 contained *bla*_IMP-4_, and 7 contained *bla*_VIM-2_. Two types of 16S-RMTases genes were detected. Eight isolates contained *armA*, while two isolates contained *rmtB*. Eight isolates coharbored *bla*_IMP-4_ and *armA* and two isolates coharbored *bla*_IMP-4_, *bla*_VIM-2_, and *armA*. No isolates harbored genes encoding AmpC β-lactamases or AMEs.

The efflux-pump gene *mexA* and the outer membrane protein gene *oprD* were detected in all 37 XDR-PA isolates. However, no mutations were detected in any of the *oprD* genes. Figure 1 lists the antibiotic resistance genes detected in each isolate.

### Expression of *mexA* and *oprD*

**Fig. 2:**
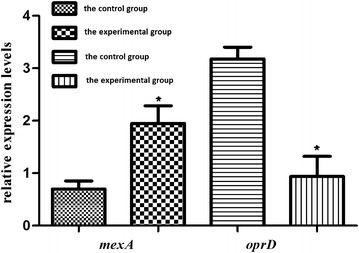
The relative expression of the *mexA* and *oprD* genes in the XDR-PA isolates. The relative expression levels are expressed as the mean ± standard error of the mean (SEM). *P values less than 0.05 are considered significant and are indicated by an *asterisk*

The expression of *mexA* was significantly higher in the 37 XDR-PA isolates than in the control group consisting of 31 antibiotic-sensitive isolates (1.95 ± 0.48 and 0.70 ± 0.13, *P* = 0.018, respectively), while expression of *oprD* in the XDR-PA strains was significantly lower than the control group (3.18 ± 0.60 and 0.94 ± 0.08; *P* = 0.002, respectively; Fig. 2).

### Distribution of genes associated with movable genetic elements

Of the movable genetic element genes tested, *intI*, *merA*, *tnp513*, *tnpA* (Tn*21*), and *tnpU* were detected in 100, 100, 81, 49, and 16 % of the isolates, respectively. Moreover, most of the 37 XDR-PA isolates coharbored three or more genes associated with movable genetic elements. For instance, isolates xy36 coharbored five types of movable genetic element genes (Fig. 1).

### Clonal relatedness of the XDR-PA isolates

Using PFGE, the 37 XDR-PA isolates were divided into 21 PFGE types (Fig. 1). The main PFGE type found was A (five strains), including three strains from Xiangya Hospital and two strains from the Third Xiangya Hospital. In addition, an A1 subtype was found in both hospitals. The B- and C-type isolates were found in the Xiangya Hospital, while the D-type isolates were found in the Third Hospital of Changsha as well as Chinese Medicine Hospital of Liuyang. The E-type strains were isolated from the Second People’s Hospital of Hunan province and the Third Xiangya Hospital, and an E1 subtype was found in both hospitals. The F-type strains were isolated from the People’s Hospital of Liuyang; the G-type strains were isolated from the Third Xiangya Hospital; and the H-type isolates were found at Xiangya Hospital and Xiangtan Central Hospital. The remaining 13 types, from I to U, were collected from the Xiangya Hospital (eight strains), the People’s Hospital of Chenzhou (one strain), the First Hospital of Changsha (one strain), the Chinese Medicine Hospital of Liuyang (one strain), the Third Xiangya Hospital (one strain), and the Third Hospital of Changsha (one strain).

## Discussion

*Pseudomonas aeruginosa* is considered one of the primary causes of hospital-acquired infections. An increase in the prevalence of clinical XDR-PA isolates correlates with a rise in mortality and morbidity rates. Consequently, XDR-PA strains pose a considerable threat to public health worldwide. In our study, 37 out of 482 (7.7 %) *P. aeruginosa* strains were found to be XDR-PA strains. The XDR-PA strains were isolated from nine of fifteen teaching hospitals detected in our region, suggesting that the occurrence of XDR-PA isolates in our region is low. To aid the prevention of the spread of XDR-PA strains, we have analyzed the resistance mechanisms and the molecular epidemiology of the XDR-PA strains.

Carbapenems are one of the most effective drugs against severe infections caused by gram-negative bacilli. Unfortunately, pathogens displaying resistance to carbapenems are increasing due to the following three main causes: production of carbapenemases, the over-expression of efflux pumps, and the diminished expression of the outer membrane porin OprD [15]. In *P. aeruginosa*, production of carbapenemases, especially MBLs, is an important antibiotic resistance mechanisms [25]. In this study, 13 (35.1 %) strains produced the metallo-enzymes IMP-4 and VIM-2, indicating that production of carbapenemases played role in carbapenem-resistant in the XDR-PA isolates. Notably, the prevalence of carbapenemases in the 37 XDR-PA isolates in our study were higher than that of other countries in the world [26, 27] and from other regions in China [28]. One isolate that coharbored two MBL genes was reported in previous studies [29]. This observation led to the emergence of a new drug-resistant model for *P. aeruginosa*. In our study, two isolates (xy35 and xy36) coharbored *bla*_IMP-4_ and *bla*_VIM-2_. To our knowledge, this is the first report of the co-existence of *bla*_IMP-4_ and *bla*_VIM-2_ in a *P. aeruginosa* strain.

Drug-efflux pumps have been correlated with bacterial resistance since the 1980s [30]. The first efflux pump found in *P. aeruginosa* is the MexAB-OprM pump, which has a broad range of substrates, including carbapenems, quinolones, aminoglycosides, tetracyclines, and macrolides. Therefore, the presence of this efflux pump in bacteria may lead to multi-drug resistance. In our study, the *mexA* gene, encoding a component of MexAB-OprM efflux pump, was found in all 37 XDR-PA isolates. Furthermore, a significant over-expression of *mexA* in the XDR-PA isolates was seen compared with the *mexA* levels in antibiotic-susceptible strains. Thus, over-expression of an efflux pump, especially MexAB-OprM, may play a key role in the antibiotic resistance of these XDR-PA isolates.

Decreased expression of OprD can significantly reduce the susceptibility of *P. aeruginosa* to carbapenems. Previous studies showed that decreased expression of *oprD* is primarily due to mutations in the OprD-encoding genes [31]. In our study, the expression of *oprD* in the 37 XDR-PA isolates was significantly lower than that in susceptible strains confirming that a decreased level of this porin plays a key role in carbapenem-resistance. However, no mutations or deletions were detected for the *oprD* gene in the 37 XDR-PA isolates. This finding was not consistent with previous studies [32, 33], suggesting that decreased expression of *oprD* in the 37 XDR-PA isolates might be due to regulation by small bioactive molecules, amino acids, or efflux pump expression [34]. Therefore, in-depth studies on the role of outer membrane proteins in bacterial drug resistance are needed in the future.

Production of AMEs and 16S-RMTases is the main cause of bacterial resistance to aminoglycoside agents [35, 36]. In contrast to these reports, none of the 37 XDR-PA isolates, which were resistant to aminoglycoside antibiotics, harbored genes encoding AMEs and only 27.0 % (n = 10) harbored either *armA* or *rmtB* 16S-RMTases genes. These data suggest that 16S-RMTases play an important role in aminoglycoside resistance in the XDR-PA isolates but also that some of these isolates may utilize an alternative, unknown resistance mechanism, such as new drug efflux pumps, new 16S-RMTases, or mutational activation of the AmgRS two-component system [37].

Movable genetic elements, including integrons, plasmids, transposons, and insertion sequences, play a key role in the horizontal transfer of resistance genes [38]. In this study, most of the XDR-PA isolates coharbored three or more genes associated with movable genetic elements. The most frequently detected genes were *intI*, *merA tnp513*, *tnpA* (Tn*21*), and *tnpU*, and their distribution percentage among the XDR-PA isolates was 100, 100, 81, 49, and 16 %, respectively. Furthermore, two isolates, cs15 and xy36, coharbored 5 types of genes associated with movable genetic elements.

The XDR-PA strains previously isolated in other countries were shown to be clonally related [1]. In contrast, the XDR-PA strains isolated from hospitals within the Hunan province of China were shown to be genetically diverse. The 37 XDR-PA isolates were divided into 21 different PFGE types and no one type predominated amongst the isolates suggesting that none of the XDR-PA isolates are considered to be an epidemic clone. Interestingly, the A-type, D-type, E-type, and H-type were detected in two different hospitals and the B-type, C-type, and F-type were found in two different wards of the same hospital during the same time period. Although prevalence of the XDR-PA isolates in this area was sporadic, dissemination of the same isolate was detected inter- and intra-hospital. The high heterogeneity of the PFGE types suggests that the mobile genetic elements may play a role in the emergence of clinical XDR-PA strains.

## Conclusions

In conclusion, this study shows that the extreme antibiotic resistance of the clinical XDR-PA isolates is due to strains coharboring multiple antibiotic resistance genes, over-expressing drug-efflux pumps, and decreasing expression of *oprD*, which encodes an outer membrane porin. Moreover, this study has identified for the first time a *P. aeruginosa* isolate that coharbors the *bla*_IMP-4_, *bla*_VIM-2_, and *armA* antibiotic resistance genes. Although the XDR-PA isolates were not clonally related, the strains were shown to carry multiple genes encoding different types of movable genetic elements, which may aid the rapid development of XDR-PA isolates.

## References

[1] Ciofi Degli Atti M, Bernaschi P, Carletti M, Luzzi I, Garcia-Fernandez A, Bertaina A, Sisto A, Locatelli F, Raponi M (2014). An outbreak of extremely drug-resistant *Pseudomonas aeruginosa* in a tertiary care pediatric hospital in Italy. BMC Infect Dis.

[2] Fonseca EL, Freitas Fdos S, Vicente AC (2010). The colistin-only-sensitive Brazilian *Pseudomonas aeruginosa* clone SP (sequence type 277) is spread worldwide. Antimicrob Agents Chemother.

[3] Jin JS, Kwon KT, Moon DC, Lee JC (2009). Emergence of *16S rRNA* methylase rmtA in colistin-only-sensitive *Pseudomonas aeruginosa* in South Korea. Int J Antimicrob Agents.

[4] Lee YC, Ahn BJ, Jin JS, Kim JU, Lee SH, do Song Y, Lee WK, Lee JC (2007). Molecular characterization of *Pseudomonas aeruginosa* isolates resistant to all antimicrobial agents, but susceptible to colistin, in Daegu, Korea. J Microbiology (Seoul, Korea).

[5] Viedma E, Juan C, Acosta J, Zamorano L, Otero JR, Sanz F, Chaves F, Oliver A (2009). Nosocomial spread of colistin-only-sensitive sequence type 235 Pseudomonas aeruginosa isolates producing the extended-spectrum beta-lactamases GES-1 and GES-5 in Spain. Antimicrob Agents Chemother.

[6] Livermore DM (2002). Multiple mechanisms of antimicrobial resistance in, *Pseudomonas aeruginosa*: our worst nightmare?. Clin Infect Dis.

[7] Poole K (2011). *Pseudomonas aeruginosa*: resistance to the max. Front Microbiol.

[8] Beige F, Baseri Salehi M, Bahador N, Mobasherzadeh S (2015). Plasmid mediated antibiotic resistance in isolated bacteria from burned patients. Jundishapur J Microbiol.

[9] Kiddee A, Henghiranyawong K, Yimsabai J, Tiloklurs M, Niumsup PR (2013). Nosocomial spread of class 1 integron-carrying extensively drug-resistant Pseudomonas aeruginosa isolates in a Thai hospital. Int J Antimicrob Agents.

[10] Stalder T, Barraud O, Casellas M, Dagot C, Ploy MC (2012). Integron involvement in environmental spread of antibiotic resistance. Front Microbiol.

[11] Aghazadeh M, Hojabri Z, Mahdian R, Nahaei MR, Rahmati M, Hojabri T, Pirzadeh T, Pajand O (2014). Role of efflux pumps: MexAB-OprM and MexXY(-OprA), AmpC cephalosporinase and OprD porin in non-metallo-beta-lactamase producing Pseudomonas aeruginosa isolated from cystic fibrosis and burn patients. Infect Genet Evol.

[12] Lee JY, KO KS (2012). OprD mutations and inactivation, expression of efflux pumps and AmpC, and metallo-beta-lactamases in carbapenem-resistant Pseudomonas aeruginosa isolates from South Korea. Int J Antimicrob Agents.

[13] Quale J, Bratu S, Gupta J, Landman D (2006). Interplay of efflux system, ampC, and oprD expression in carbapenem resistance of Pseudomonas aeruginosa clinical isolates. Antimicrob Agents Chemother.

[14] Xavier DE, Picao RC, Girardello R, Fehlberg LC, Gales AC (2010). Efflux pumps expression and its association with porin down-regulation and beta-lactamase production among Pseudomonas aeruginosa causing bloodstream infections in Brazil. BMC Microbiol.

[15] Castanheira M, Deshpande LM, Costello A, Davies TA, Jones RN (2014). Epidemiology and carbapenem resistance mechanisms of carbapenem-non-susceptible Pseudomonas aeruginosa collected during 2009–11 in 14 European and Mediterranean countries. J Antimicrob Chemother.

[16] Polotto M, Casella T, de Lucca Oliveira MG, Rubio FG, Nogueira ML, de Almeida MT, Nogueira MC (2012). Detection of *P. aeruginosa* harboring bla CTX-M-2, bla GES-1 and bla GES-5, bla IMP-1 and bla SPM-1 causing infections in Brazilian tertiary-care hospital. BMC Infect Dis.

[17] Clinical and Laboratory Standards Institute. Performance standards for antimicrobial susceptibility testing, 24th informational supplement. M100-S24. Wayne: Clinical and Laboratory Standards Institute; 2014.

[18] Fang H, Ataker F, Hedin G, Dornbusch K (2008). Molecular epidemiology of extended-spectrum beta-lactamases among Escherichia coli isolates collected in a Swedish hospital and its associated health care facilities from 2001 to 2006. J Clin Microbiol.

[19] Perez-Perez FJ, Hanson ND (2002). Detection of plasmid-mediated AmpC beta-lactamase genes in clinical isolates by using multiplex PCR. J Clin Microbiol.

[20] Queenan AM, Bush K (2007). Carbapenemases: the versatile beta-lactamases. Clin Microbiol Rev.

[21] Soleimani N, Aganj M, Ali L, Shokoohizadeh L, Sakinc T (2014). Frequency distribution of genes encoding aminoglycoside modifying enzymes in uropathogenic *E. coli* isolated from Iranian hospital. BMC Res Notes.

[22] Doi Y, Arakawa Y (2007). Ribosomal RNA methylation: emerging resistance mechanism against aminoglycosides. Clin Infect Dis.

[23] Arabestani MR, Rajabpour M, Yousefi Mashouf R, Alikhani MY, Mousavi SM. Expression of efflux pump MexAB-OprM and OprD of Pseudomonas aeruginosa strains isolated from clinical samples using qRT-PCR. Arch Iranian Med. 2015;18(2):102–8. doi:015182/AIM.008. 25644798

[24] Tenover FC, Arbeit RD, Goering RV, Mickelsen PA, Murray BE, Persing DH, Swaminathan B (1995). Interpreting chromosomal DNA restriction patterns produced by pulsed-field gel electrophoresis: criteria for bacterial strain typing. J Clin Microbiol.

[25] Hong DJ, Bae IK, Jang IH, Jeong SH, Kang HK, Lee K (2015). Epidemiology and Characteristics of Metallo-β-Lactamase-Producing *Pseudomonas aeruginosa*. Infect Chemother..

[26] Kalantar E, Torabi V, Salimizand H, Soheili F, Beiranvand S (2012). Soltan Dallal MM. First survey of metallo-β-lactamase producers in clinical isolates of *Pseudomonas aeruginosa* from a referral burn center in Kurdistan Province. Jundishapur J Nat Pharm. Prod..

[27] Hong JS, Kim JO, Lee H, Bae IK, Jeong SH, Lee K (2015). Characteristics of metallo-β-lactamase-producing *Pseudomonas aeruginosa* in Korea. Infect Chemother..

[28] Zhang X, Gu B, Mei Y, Wen Y, Xia W (2015). Increasing resistance rate to carbapenem among blood culture isolates of *Klebsiella pneumoniae*, *Acinetobacter baumannii* and *Pseudomonas aeruginosa* in a university-affiliated hospital in China, 2004–2011. J Antibiot (Tokyo)..

[29] Toval F, Guzmán-Marte A, Madriz V, Somogyi T, Rodríguez C, García F (2015). Predominance of carbapenem-resistant *Pseudomonas aeruginosa* isolates carrying *bla*_IMP_ and *bla*_VIM_ metallo-β-lactamases in a major hospital in Costa Rica. J Med Microbiol..

[30] George AM, Levy SB (1983). Amplifiable resistance to tetracycline, chloramphenicol, and other antibiotics in *Escherichia coli*: involvement of a non-plasmid-determined efflux of tetracycline. J Bacteriol..

[31] Shu JC, Chia JH, Siu LK, Kuo AJ, Huang SH, Su LH, Wu TL (2012). Interplay between mutational and horizontally acquired resistance mechanisms and its association with carbapenem resistance amongst extensively drug-resistant *Pseudomonas aeruginosa* (XDR-PA). Int J Antimicrob Agents..

[32] Shen J, Pan Y, Fang Y (2015). Role of the outer membrane protein OprD2 in carbapenem-resistance mechanisms of *Pseudomonas aeruginosa*. PLoS One..

[33] Rojo-Bezares B, Estepa V, Cebollada R, de Toro M, Somalo S, Seral C, Castillo FJ, Torres C, Sáenz Y (2014). Carbapenem-resistant *Pseudomonas aeruginosa* strains from a Spanish hospital: characterization of metallo-beta-lactamases, porin OprD and integrons. Int J Med Microbiol..

[34] Li H, Luo YF, Williams BJ, Blackwell TS, Xie CM (2012). Structure and function of OprD protein in *Pseudomonas aeruginosa*: from antibiotic resistance to novel therapies. Int J Med Microbiol..

[35] Ashenafi M, Ammosova T, Nekhai S, Byrnes WM (2014). Purification and characterization of aminoglycoside phosphotransferase APH(6)-Id, a streptomycin-inactivating enzyme. Mol Cell Biochem..

[36] Galani I, Souli M, Panagea T, Poulakou G, Kanellakopoulou K, Giamarellou H (2012). Prevalence of 16S rRNA methylase genes in *Enterobacteriaceae* isolates from a Greek university hospital. Clin Microbiol Infect..

[37] Lau CH, Fraud S, Jones M, Peterson SN, Poole K (2013). Mutational activation of the AmgRS two-component system in aminoglycoside-resistant *Pseudomonas aeruginosa*. Antimicrob Agents Chemother..

[38] Ibrahim ME, Magzoub MA, Bilal NE, Hamid ME (2013). Distribution of Class I integrons and their effect on the prevalence of multi-drug resistant *Escherichia coli* clinical isolates from Sudan. Saudi Med J..

